# HIV Infection Complicated with Cytomegalovirus Colitis: A Case Report of ^18^F-FDG PET/CT Imaging

**DOI:** 10.2174/0115734056361753241226065721

**Published:** 2025-01-03

**Authors:** Peipei Zhang, Shengwei Fang

**Affiliations:** 1 Department of Nuclear Medicine, Hangzhou Cancer Hospital, Hangzhou 310002, China

**Keywords:** Cytomegalovirus, Human immunodeficiency virus, Acquired immune deficiency syndrome, Colitis, 18F-FDG, PET/CT, Case report

## Abstract

**Background:**

Cytomegalovirus (CMV) infection is common in the digestive and central nervous systems and can infect the entire digestive tract from the mouth to the rectum. In immunocompromised patients, CMV infection is prone to develop into CMV disease, especially in Acquired Immune Deficiency Syndrome (AIDS) patients. Severe cases may accelerate the progression of AIDS patients and form systemic CMV infection. Timely diagnosis and treatment are very important for the prognosis of patients.

**Case Presentation:**

In this paper, we report a 36-year-old man with a Human Immunodeficiency Virus (HIV) infection complicated with CMV colitis. Three weeks ago, he developed abdominal pain with fresh blood in the stool, accompanied by anal pain. He was found to be HIV positive 8 years ago. An enhanced CT scan showed edema and irregular thickening of the rectal wall, obvious enhancement of the mucosa, and multiple enlarged lymph nodes around. ^18^F-FDG PET/CT imaging displayed diffuse rectum wall thickening and increased glucose metabolism, and the SUV max was 12.7. There were multiple enlarged lymph nodes around the rectum, glucose metabolism was increased, and the SUVmax was 4.6.

**Conclusion:**

^18^F-FDG-PET imaging technology has potential value in the diagnosis of CMV colitis, especially in immunocompromised patients. Detection of FDG concentrations in the colon wall can help diagnose CMV infection and understand the extent of the lesion, which is essential for the timely initiation of antiviral therapy.

## 1 INTRODUCTION

Cytomegalovirus (CMV) is a pathogen that causes infected cells to enlarge and exhibit large intranuclear inclusion bodies. It is ubiquitous in nature. The CMV that infects humans is known as Human Cytomegalovirus (HCMV), also referred to as Human Herpesvirus 5(HHV-5), belonging to the subfamily of Herpesvirus B subfamily [1]. After infection, it remains in the body for life. Almost all adults have CMV infection, with a seroprevalence rate of 40-100%, but it is generally asymptomatic [1, 2]. Individuals with Acquired Immune Deficiency Syndrome (AIDS) represent the primary population affected by CMV infection, which can involve the entire gastrointestinal tract, from the oral cavity to the anus; however, the colon and rectum are the most frequently involved sites, accounting for 47% of cases [2]. CMV gastrointestinal infection mainly manifests as anorexia, fever, diarrhea, bloody stools, tenesmus, abdominal pain, *etc*. The pathology can range from mild inflammation to ulcers. CMV-related gastrointestinal diseases are caused by vascular inflammation in the submucosa of the intestines, which can lead to thrombosis, and ischemia, resulting in intestinal ulcers, intestinal wall thickening, and occasionally gangrene or perforation [3]. Infection with CMV can lead to T cell subset dysfunction or even failure [4, 5]. In severe cases, this may accelerate the progression of conditions in AIDS patients, culminating in systemic CMV infection, which is a significant contributor to mortality among this population [6].

## 2 CASE DESCRIPTION

The procedures outlined in this report adhere to established ethical standards set forth by the institutional research committee of Hangzhou Cancer Hospital. An informed consent form was signed by the patient. He was a 36-year-old male. He identified as homosexual and had engaged in anal intercourse for over ten years. He was found to be Human Immunodeficiency Virus (HIV) positive 8 years ago, with a CD4+ count of 700 cells/μL at that time. He started treatment with Biktarvy (Bictarvy, Bictegravir tablets) in July 2022(2 years ago). He was diagnosed with syphilis 2 years ago and treated with long-acting penicillin at that time. Currently, his CD4+ count was 1400 cells/μl, his syphilis TRUST was positive at a titer of 1:1, and TPPA was positive. He had no history of hypertension, diabetes, smoking, alcohol consumption and food or drug allergies. There was nothing unusual in the physical examination. Three weeks prior to admission, the patient experienced abdominal pain accompanied by fresh blood in the stool, with no identifiable cause. This was also associated with anal pain that did not alleviate after defecation. The physical examination revealed no abnormalities. After admission, routine blood tests, liver, and kidney function, electrolytes, high-sensitivity C-reactive protein, and male tumor markers were all within normal limits.. The blood CMV-DNA level was 1560 copies/mL. Immunoglobulin IgG, IgM, IgA, C3, and C4 levels were normal. The absolute count of helper/induced T lymphocytes (D3+, CD4+) was 1399 cells/μL (reference range: 441-2156 cells/μL). Fungal D-glucan test indicated a 1,3-β-D-glucan level of <10.00 pg/mL (reference range: <60 pg/mL). Cryptococcal antigen test (blood) was negative. Tuberculosis infection T-cell test (blood) (chemiluminescence method) was negative.

**Fig. (1):**
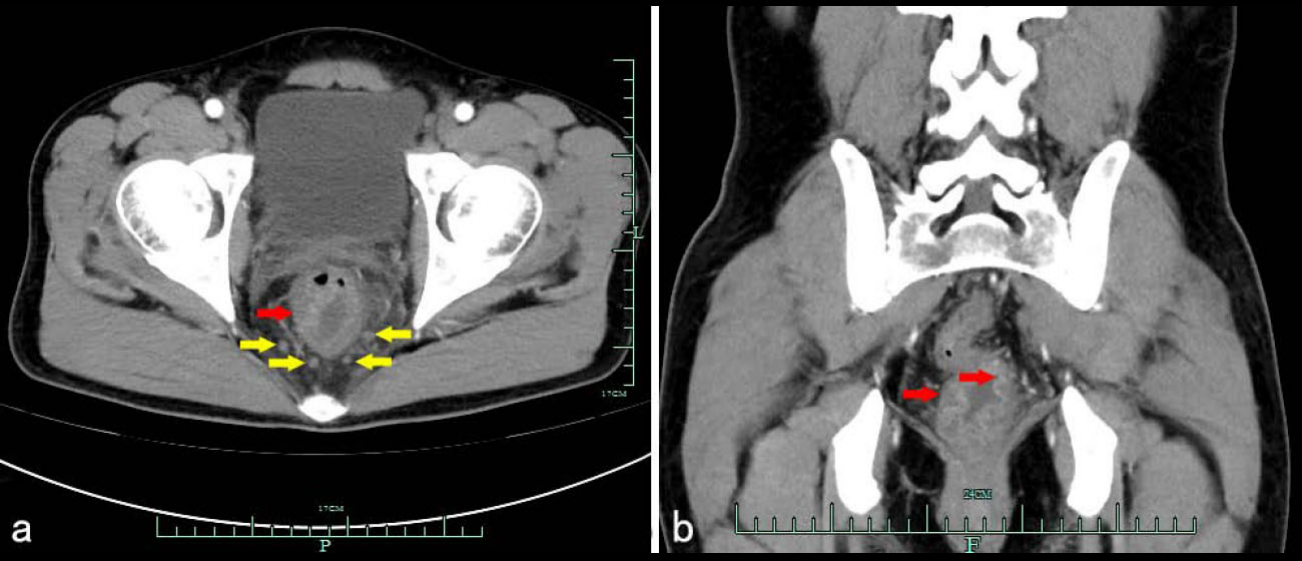
Pelvic Enhance CT in patients with CMV colitis: (**a**) axial scan; (**b**) coronal scan. CT scan showed Edema and irregular thickening of the rectal wall (red arrow), obvious enhancement of the mucosa (red arrow), and multiple enlarged lymph nodes around (yellow arrow).

**Fig. (2):**
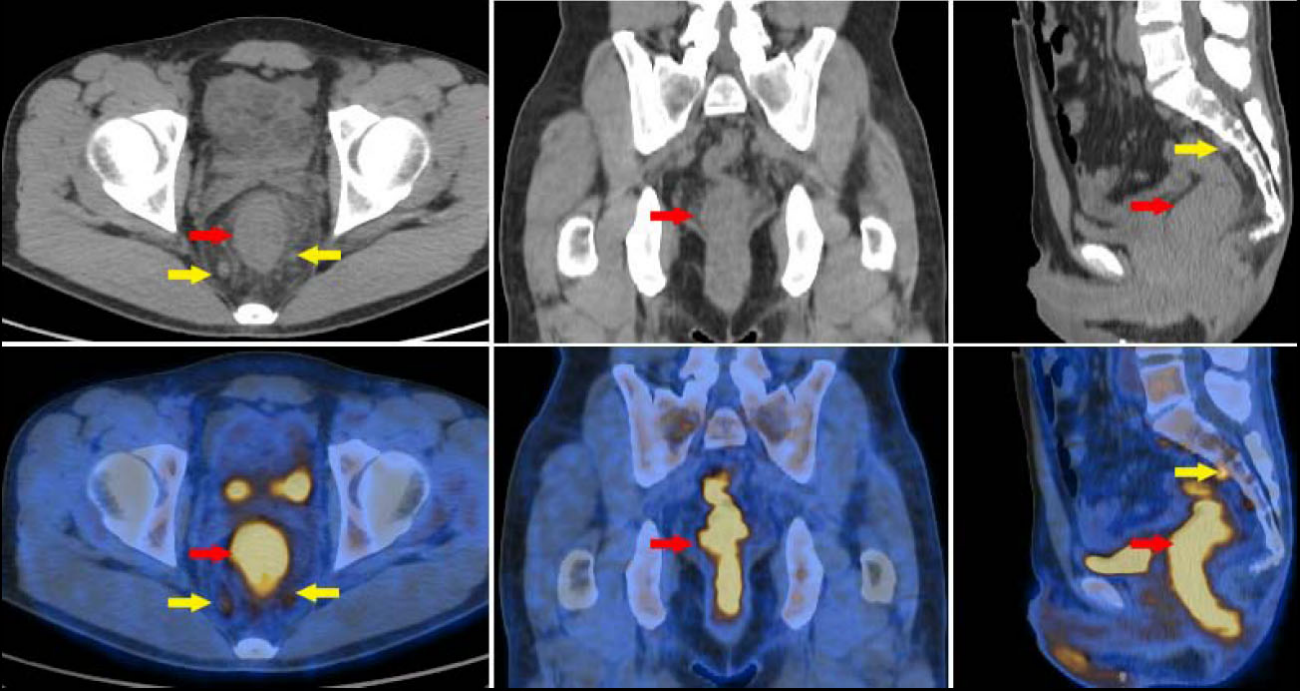
^18^F-FDG PET/CT imaging in patients with CMV colitis. PET/CT imaging displayed diffuse rectum wall thickening and increased glucose metabolism,and the SUVmax was 12.7(red arrow). There were multiple enlarged lymph nodes around the rectum, some of them were enlarged, the short diameter of the larger one was 0.8cm, glucose metabolism was increased, and the SUVmax was 4.6(yellow arrow).

Enhanced CT scan showed edema and irregular thickening of the lower rectal wall, obvious enhancement of the mucosa, and multiple enlarged lymph nodes around, and the preliminary CT diagnosis was proctitis (Fig. **1**). The patient fasted for 8 hours prior to the ^18^F-FDG PET/CT examination. The patient's blood glucose level was measured at 98 mg/dL before the injection of ^18^F-FDG. Following the intravenous administration of ^18^F-FDG (produced by Shanghai Atom Science Pharmaceutical Co., Ltd., with a radiochemical purity exceeding 95%) at a dosage of 3.3 MBq/kg, imaging was performed 60 minutes later using the GE Discovery PET/CT 710. The parameters for the spiral CT scanning were as follows: voltage of 120 kV, tube current of 150 mA, and slice thickness of 3.75 mm. The parameters for the PET scanning included a slice thickness of 3 mm, a field of view of 600 mm, 8 beds, and a duration of 3 minutes per bed. CT images were reconstructed using a standard method, while PET images were reconstructed using the OSEM method and corrected for attenuation based on the CT data. PET/CT imaging demonstrated diffuse thickening of the rectal wall and anal canal, which was associated with elevated glucose metabolism, as indicated by a maximum standardized uptake value (SUVmax) of 12.7. The adipose tissue surrounding the lesion exhibited a blurred appearance. Additionally, multiple enlarged lymph nodes were identified in the perirectal region. Among these, several nodes were notably enlarged, with the largest measuring a short diameter of 0.8 cm and demonstrating increased glucose metabolism, as indicated by a SUVmax of 4.6 (Fig. **2**). In comparison to enhanced CT, PET/CT identified a broader spectrum of lesions, encompassing nearly the entire rectum and anal canal.

**Fig. (3):**
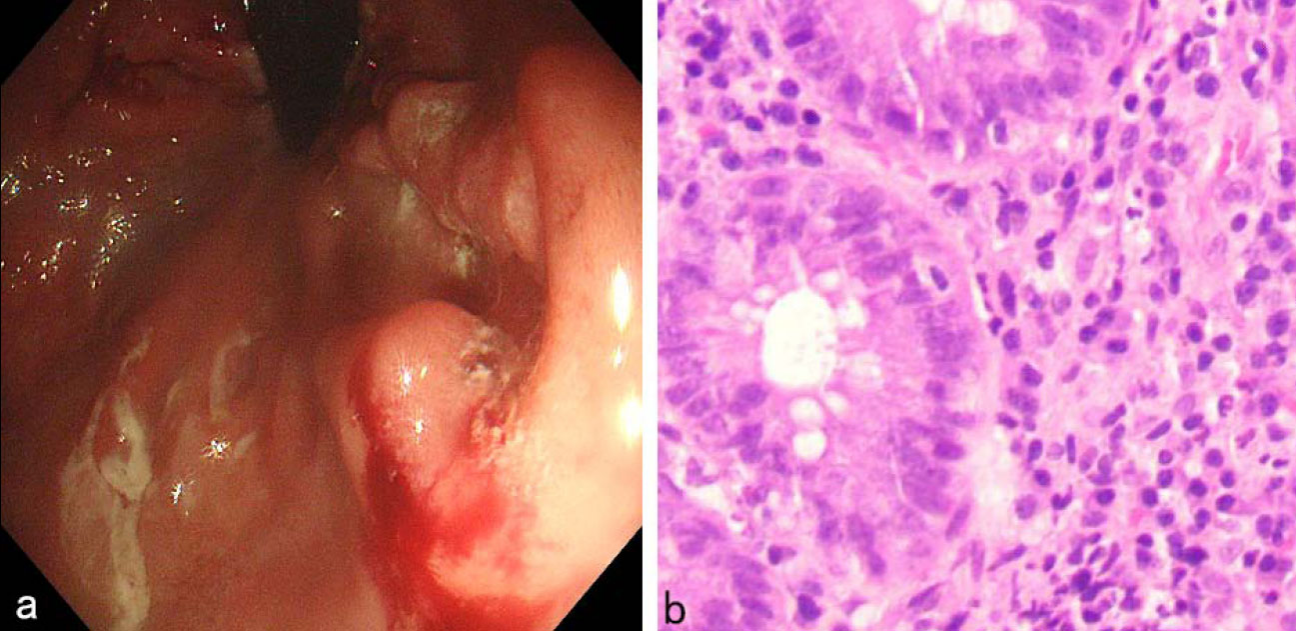
(**a**) Colonoscopy in patients with CMV colitis: Colonoscopy showed ulcers of varying sizes in the colon, covered with yellow moss, and elevated mucosa around; (**b**) HE staining in patients with CMV colitis(×200): HE staining displayed: “Rectum” mucosa chronic active inflammation with erosion, granulation tissue hyperplasia, a few crypt branches, individual crypt abscesses, a small area of reduced mucus secretion, large amounts of lymphocytes, plasma cells infiltrating the mucosa and submucosa.

Colonoscopy showed ulcers of varying sizes in the colon, covered with yellow moss, and elevated mucosa around (Fig. **3a**); Hematoxylin-eosin (HE) staining displayed: “Rectum” mucosa chronic active inflammation with erosion, granulation tissue hyperplasia, a few crypt branches, individual crypt abscesses, a small area of reduced mucus secretion, large amounts of lymphocytes, and plasma cells infiltrating the mucosa and submucosa (Fig. **3b**). HE staining indicated the presence of inflammatory lesions in the rectum. The findings from the Immunohistochemistry (IHC) analysis are summarized as follows: CD3 partially (+), CD5 partially (+), PAX5 partially (+), CD20 partially (+), CD19 partially (+), MUM1 plasma cells (+), CD138 plasma cells (+), Bcl-2 partially (+), CD68 histiocytes (+), CD21 a few FDC nets, Ki-67 (15%+), tuberculosis PCR (-), acid-fast (-), silver staining (-), mucicarmine (-), PAS (+), TB fluorescence (-), fungal immunofluorescence (-), CMV2 (a small amount +), and EBER (-). IHC suggested a slightly increased number of B cells, predominantly plasma cells, without any significant abnormal distribution of T and B cells. The pathological conclusion indicated chronic inflammation in the rectal mucosa (active), characterized by erosion, necrosis, and hyperplasia of granulation tissue, consistent with ulcerative changes. This was accompanied by an aggregation of interstitial lymphocytes, plasma cells, and histiocytes, as well as a small number of cells indicative of CMV infection.

According to relevant consensus, and considering that the patient's current HIV infection is well-controlled and the lesions associated with CMV infection are relatively localized, a reduced dosage of ganciclovir injection therapy is being administered [2-4, 6]. Ganciclovir 250 mg was administered intravenously every 12 hours for the treatment of CMV infection, with a total treatment duration of three weeks. The patient's abdominal pain with fresh blood in the stool and anal pain disappeared. The re-examination of CMV DNA turned negative. No adverse or unanticipated events occurred during the diagnosis and treatment process.

## 3 DISCUSSION

CMV infection is common in the digestive and central nervous systems and can infect the entire gastrointestinal tract, extending from the oral cavity to the rectum. The clinical manifestations primarily include fever, abdominal pain, diarrhea, and fatigue. Colonoscopy can show large areas of ulcers and, in severe cases, can see hemorrhagic erosion and diffuse erosions, which is not easy to distinguish from Inflammatory Bowel Disease (IBD) [7, 8]. In patients with AIDS, CMV infection is common in the eyes, digestive system, and central nervous system. The incidence and severity of CMV infection-related digestive system diseases are related to cellular immune function. In CMV infection-related digestive system diseases, the most commonly affected sites are the esophagus and colon, with colitis being the most common [6, 9]. Characteristic findings during colonoscopy include longitudinal ulcers, deep chisel-like ulcers, and cobblestone signs [10].

For immunocompromised individuals, CMV infection is prone to develop into CMV disease. This is particularly evident in patients with AIDS who exhibit CD4+ T cells <100 cells/μL and HIV RNA >100,000 copies/mL, as they are more likely to develop CMVD [5]. In clinical practice, AIDS patients with low CD4+ T cell counts should be screened for CMV infection. Although the patient described in this case report has been living with HIV for over eight years, the probability of direct CMV infection remains low due to the elevated levels of CD4+ T cell counts achieved through standard antiretroviral therapy. Furthermore, the infection may also be associated with rectal mucosal injury resulting from anal intercourse.

HE staining in histological analysis can be utilized to diagnose CMV infection by identifying inclusion bodies within the nuclei of giant cells, exhibiting a specificity ranging from 92% to 100%. However, this method is characterized by low sensitivity, which often leads to false-negative results. In contrast, the IHC detection of CMV antigens exhibits a higher sensitivity, reported to be between 78% and 93%, compared to conventional HE staining [2, 11]. Consequently, this technique improves the detection rate of CMV and is currently considered the gold standard for diagnosing CMV infection. Also, the sensitivity and specificity for the cutoff value of tissue CMV DNA were 78.9 and 74.3% [11].

CMV infection should be treated with valganciclovir or intravenous ganciclovir; the preferred treatment plan for CMV colitis patients is ganciclovir 5mg/(kg·time), intravenous infusion, once every 12 hours, for a course of ≥3 weeks, until symptoms and signs disappear, and maintenance treatment is generally not recommended [2, 6, 12].

The patient reported in this case has a documented history of AIDS and syphilis spanning several years, with the primary symptoms being abdominal pain and diarrhea. Enhanced CT indicated inflammatory lesions in the lymph nodes and rectum. However, identifying a specific etiology proved to be challenging. PET/CT indicated that the lesions were localized to the rectum and anal canal, characterized by thickening of the intestinal wall, obscuration of the surrounding fat space, multiple lymphadenopathies, partial swelling, and increased glucose metabolism. No significant abnormal lesions were identified in other segments of the gastrointestinal tract. Based on the findings from the PET/CT scan, inflammatory lesions were prioritized in the differential diagnosis. The most prevalent intestinal inflammatory diseases include IBD (ulcerative colitis, Crohn's disease) and infectious pathogens. Ulcerative colitis and Crohn's disease are classified as chronic, non-specific inflammatory bowel diseases, typically presenting with recurrent abdominal pain, diarrhea, and mucopurulent bloody stools. Ulcerative colitis primarily affects the mucosa and submucosa of the large intestine, predominantly in the sigmoid colon and rectum, with potential extension to the descending colon or the entire colon. In contrast, Crohn's disease primarily involves the ileum, cecum, and the right colon. Given the lesions observed on the PET/CT, both ulcerative colitis and Crohn's disease can be excluded from consideration. Common infectious pathogens in patients with AIDS include syphilis, tuberculosis, fungi, bacteria, CMV, and Epstein-Barr Virus (EBV). Tuberculous colitis typically occurs in the cecum or the terminal ileum; however, the lesions identified in this case suggest that tuberculous colitis is unlikely. Although the patient has a history of syphilis, the TRUST titer was recorded at 1:1 post-treatment, effectively ruling out the possibility of syphilis recurrence affecting the rectum. The identification of other pathogens remains complex, as clinical symptoms often lack distinctive features, and there are limited laboratory tests with diagnostic significance, leading to a high likelihood of missed or misdiagnosed cases. A comprehensive analysis utilizing etiological, pathological, and immunological methods is essential for accurate diagnosis. Blood tests did not provide evidence of tuberculosis or fungal infections. IHC results indicated negative findings for tuberculosis fluorescence, tuberculosis PCR, and acid-fast bacilli, thereby excluding tuberculosis infection. Additionally, silver staining, mucicarmine staining, and fungal immunofluorescence tests were negative, ruling out fungal and bacterial infections. EBER testing was also negative, excluding EBV infection. Ultimately, due to the positive result for CMV, the final diagnosis was established as CMV colitis.

At present, as far as we know, there are only two literature reports on joint CMV colitis ^18^FDG PET or PET/CT imaging (Table **1**). Both documents discuss the application of ^18^F-FDG PET imaging technology in the diagnosis of CMV colitis.

Takashi Nihashi reported a case of a 63-year-old female patient who had a fever of unknown origin and watery diarrhea due to brain consciousness disturbance (caused by moyamoya disease) [13]. Through ^18^F-FDG-PET, colonoscopy, blood analysis, and CMV antigenemia determination, FDG was found to be concentrated in the dilated colon wall, extending from the transverse colon to the sigmoid colon. Colonoscopy showed edematous, inflammatory damage, and perforating ulcers consistent with the area of abnormal FDG uptake. CMV antibodies were detected in the colonic mucosa of the biopsy samples, and CMV antigenemia was detected by immunohistochemical analysis using monoclonal antibodies against CMV pp65 antigen. Based on these findings, CMV colitis was strongly suspected, and treatment with ganciclovir was initiated, followed by relief of colitis symptoms.

Anna Sophie L. Kjaer reported a case of a patient with IBD treated with azathioprine who developed acute CMV infection and CMV colitis [14]. The patient underwent an ^18^F-FDG PET-CT examination two weeks after the onset of symptoms, and the results showed pathological ^18^F-FDG uptake in the mucosa of the left colon, similar to the activity of IBD. The diagnosis of CMV colitis was based on the presence of CMV IgM antibodies, seroconversion of CMV IgG antibodies, the presence of CMV DNA in the plasma, and the detection of CMV DNA in colonic mucosa biopsy. The patient responded well to ganciclovir treatment. This case emphasizes that a positive scan of the colon on ^18^F-FDG PET-CT may be caused by CMV colitis.

Patients with AIDS are particularly susceptible to opportunistic infections, which can be identified through imaging modalities such as CT, MRI, and ultrasound at the onset of these infections. However, the time required for significant pathological anatomical changes may result in the absence of detectable lesions during the early stages of infection, leading to the potential oversight of early inflammatory and infectious lesions, as well as partially occult lesions. Focal inflammation and infection can stimulate the activation of various white blood cells, including granulocytes, monocytes, and lymphocytes, which subsequently release interleukins that upregulate glucose transporters. Both acute and chronic inflammatory and infectious processes can enhance FDG uptake, thereby facilitating detection through PET/CT imaging. Furthermore, PET/CT provides a comprehensive assessment of the entire body, which is advantageous for diagnostic purposes and for evaluating the overall condition of the patient. It can also assist in localizing pathogens or guiding tissue biopsies. The diagnostic evaluation in this case illustrates that PET/CT, due to its superior sensitivity, can more accurately delineate the extent of intestinal lesions compared to conventional CT, thereby offering enhanced support for diagnosis, differential diagnosis, and treatment planning [15, 16]. Artificial intelligence technology is increasingly being integrated into medical imaging and diagnostic workflows, encompassing various stages from image acquisition and data analysis to differential diagnosis [17-20]. The rapid advancements in artificial intelligence applications in PET/CT are significantly enhancing the diagnostic efficiency of PET/CT in the assessment of inflammatory conditions [21].

## CONCLUSION

^18^F-FDG-PET/CT imaging technology has potential value in the diagnosis of CMV colitis, especially in immunocompromised patients. Detection of FDG concentrations in the colon wall can help diagnose CMV infection and understand the extent of the lesion, which is essential for the timely initiation of antiviral therapy. Furthermore, ^18^F-FDG-PET/CT provides a comprehensive assessment of the entire body, which is advantageous for diagnostic purposes and for evaluating the overall condition of the patient with AIDS. It can also assist in localizing pathogens or guiding tissue biopsies.

## Figures and Tables

**Table 1:** Previously reported cases of CMV colitis using ^18^F-FDG PET imaging.

**Author/Refs.**	**Published Time**	**Age**	**Gender**	**Location**	**Underlying Disease**	**SUVmax**
Takashi Nihashi [13]	2006	63	Male	The left side of the colon	HIV(-)	5.0
Anna Sophie L. Kjaer [14]	2019	33	Male	The left side of the colon	HIV(-)、IBD	NA

## Data Availability

The corresponding author will provide access to the requested information upon reasonable request.

## LIST OF ABBREVIATIONS

CMV: Cytomegalovirus
HCMV: Human Cytomegalovirus
HHV-5: Human Herpesvirus 5
AIDS: Acquired Immune Deficiency Syndrome
HIV: Human Immunodeficiency Virus
HE: hematoxylin-eosin
IHC: immunohistochemistry
IBD: Inflammatory Bowel Disease,

## STANDARDS OF REPORTING

CARE guidelines were followed.
